# Plasma Proteomic Profile of Chemotherapy‐Induced Severe Neutropenia: A Pilot Discovery Phase Study

**DOI:** 10.1096/fj.202503947RR

**Published:** 2026-02-01

**Authors:** Leticia Queiroz da Silva, Alexander Leonardo Silva‐Junior, Licia C. Silva‐Costa, Ivanio Teixeira Borba‐Junior, Bradley J. Smith, Edilson Tadeu Andrade, Bruno Kosa Lino Duarte, Marcos Paulo Colella, Thiago Martins Santos, Daniel Martins‐de‐Souza, Erich Vinicius De Paula

**Affiliations:** ^1^ School of Medical Sciences University of Campinas Campinas SP Brazil; ^2^ Institute of Biology University of Campinas Campinas SP Brazil; ^3^ Hematology and Hemotherapy Center University of Campinas Campinas SP Brazil

**Keywords:** biomarkers, drug therapy, hematologic cancer, immunothrombosis, sepsis, system biology

## Abstract

Transient severe neutropenia and thrombocytopenia are frequent during treatment of hematological malignancies and contribute to early mortality through sepsis and bleeding. While neutrophils and platelets are essential for host defense and tissue repair, little is known about immune adaptations during their depletion. In this pilot, discovery phase study, we evaluated the plasma proteome of 10 patients with chemotherapy‐induced severe neutropenia and thrombocytopenia, prior to any clinical complication. At sampling, mean neutrophil and platelet counts were 0.15 ± 0.18 × 10^9^/L and 34.6 ± 17.4 × 10^9^/L, respectively. A total of 762 proteins were identified, of which 71 were downregulated and 21 were upregulated in patients compared with healthy controls. Over‐representation analyses revealed that most downregulated proteins were related to immune responses, hemostasis, and protein metabolism. Notably, six upregulated proteins (USP8, IGFBP2, RCN1, B2M, LRG1, and C9) also mapped to these pathways, supporting the hypothesis that they may contribute to early systemic responses to chemotherapy‐induced myelotoxicity and tissue damage. This exploratory study leveraged a unique clinical window to examine how the human immune system responds to the marked reduction of neutrophils and platelets in the context of tissue and barrier disruption. The downregulation profile suggests impairment of the host's immunothrombotic defense, whereas the upregulated proteins may represent early compensatory mechanisms aimed at preserving homeostasis. Together, these findings provide insights into systemic responses during profound cytopenias and highlight candidate proteins that may mediate early adaptations to hematopoietic and tissue injury in patients undergoing intensive chemotherapy.

## Graphical abstract

Immune system, hemostasis, and protein metabolism are the major biological mechanistic effects affected by chemotherapy‐induced myelotoxicity treatment, which may underline tissue damage and systemic responses seen in patients' follow‐up treatment.

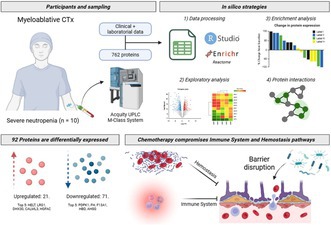

## 1 Introduction

Patients with hematological malignancies that require myeloablative chemotherapy for their treatment or as part of hematopoietic stem cell transplantation (HSCT) commonly develop severe neutropenia and thrombocytopenia, associated with widespread disruption of epithelial barriers in the gastrointestinal tract [1]. This transient state renders patients more prone to sepsis and bleeding complications, representing a major challenge in the treatment of hematological malignancies [2, 3].

Neutrophils and platelets are recognized as key elements of the host response to pathogens, as part of a process known as immunothrombosis, which consists of the localized activation of hemostasis and innate immunity triggered by molecules released from pathogens or from sterile tissue damage [4]. The concomitant activation of innate immunity and hemostasis contributes to pathogen containment and eradication as part of a beneficial biological response but can also mediate tissue damage when activated in a dysregulated and nonlocalized fashion [5, 6, 7]. Immunothrombosis pathways have been increasingly recognized as both beneficial and detrimental during sepsis and COVID‐19 [8, 9, 10]. Neutrophil components, such as free DNA and histones, induce thrombin generation and drive coagulation activation inside blood vessels [11]. Platelets, on the other hand, were shown to contribute to both immune system activities [12, 13] and cell‐to‐cell aggregation [5, 14], mainly by mechanisms of recognition and prompt both defense responses to pathogens and immune cell migration to tissues [15]. Therefore, these findings support the concept that these cells shape the immunothrombotic response.

A transient period of severe neutropenia and thrombocytopenia is expected as part of the induction phase of the treatment of hematological malignancies or HSCT, thus representing an attractive human model to understand how pathways involved in the host response adapt to the absence of their major actors, as well as the consequences of the widespread breakdown of tissue barriers observed after intensive chemotherapy [1].

To gain insights on the impact of major decrease of circulating neutrophils and platelets on the human proteome, we designed a pilot study that compared the plasma proteomic profile of healthy individuals and onco‐hematological patients with chemotherapy‐induced severe neutropenia prior to the development of any complication.

## 2 Materials and Methods

### 2.1 Study Population

Our study was conducted in an academic tertiary hospital in Brazil. Inclusion criteria were: (i) diagnosis of onco‐hematological malignancy, presenting with chemotherapy‐induced severe neutropenia (absolute neutrophil count < 0.5 × 10^3^/μL) without fever (*T* < 37.5°C) and age from 18 to 70 years old. Exclusion criteria were recent surgery, trauma, hemorrhagic or cardiogenic shock. The inclusion and exclusion criteria were designed to select a study population characterized by chemotherapy‐induced severe neutropenia and thrombocytopenia, irrespective of baseline diagnosis. A control group of healthy individuals (HI) with the same age range with no acute or chronic disease in the 2 weeks prior to recruitment was also included. All participants provided written informed consent, and the study followed the Helsinki Declaration. This research was approved by Institutional Review Board Statement (IRB) from University of Campinas and Hospital das Clínicas ethical committee prior to patients' recruitment under IRB approval of #5.581.845.

### 2.2 Sample and Data Collection

Samples were obtained around the nadir of neutrophil counts following a recent cycle of myeloablative chemotherapy. After these cycles, complete blood counts are obtained every 2–3 days to provide transfusion support, and patients were included in our study at the first time‐point in which the inclusion criteria were fulfilled. Both outpatients and inpatients were screened and invited to participate. Peripheral whole blood was obtained by venipuncture, collected in tubes with sodium heparin for plasma obtention. Samples were maintained on ice up to 2 h after collection, and centrifuged at 1500 *g*, 4°C, 10 min; plasma aliquots were stored at −80°C for proteomic assays. Clinical and demographic data were obtained from patient medical records and stored in REDCap (RRID:SCR_003445), a secure web‐based capture system.

This study was designed as a discovery‐phase proteomic analysis. Sample size was determined based on analytical feasibility and best practices in mass spectrometry–based proteomics, prioritizing sample quality and minimization of batch effects. A group of 10 patients was selected using predefined clinical and laboratory criteria aimed to capture the biological variability of chemotherapy‐induced severe neutropenia, capable of identifying differences of 1.25 standard deviations with a statistical power of 0.8 and a significance level of 0.05.

### 2.3 Proteomic Analysis

Heparin plasma was processed by a locally established protocol. Proteins were identified and quantified by bottom‐up shotgun mass spectrometry. First, immunodepletion of specific proteins with high serum abundance was performed by High Performance Liquid Chromatography (HPLC) using a Human‐14 Multiple Affinity Removal Column (5188‐6558, Agilent Technologies). The depleted proteome was quantified using Pierce BCA Protein Assay Kits (ref. 23225, ThermoFisher Scientific). Samples were prepared for LC–MS/MS analysis using the filter‐aided sample preparation method [16]. Briefly, 50 μg of protein were digested with modified porcine trypsin (V5111, Promega) with a ratio of 1:50 (trypsin: total protein). Proteins were reduced, alkylated, and digested on 10 kDa filters. Peptides were eluted, lyophilized, and stored at −80°C before being resuspended in 0.1% aqueous formic acid with brief vortexing and bath sonication for 10 min to a concentration of 0.25 μg/μL before injection.

Using an M‐Class UPLC system (Waters Corporation), 1 μg of peptides was loaded onto a C18 trap column (Waters Corporation; 100A, 5 μm, 180 μm × 20 mm) for 10 min at a flow rate of 8 μL/min before being eluted onto an HSS T3 analytical column (Waters Corporation; 1.8um, 75umx150mm) held at 40°C over a 90‐min gradient from 3% solvent B (0.1% formic acid [Honeywell] in LC–MS hypergrade acetonitrile [Supelco]) to 40% solvent B before eluting at 85% solvent B for 10 min and returning to 3% solvent B to reequilibrate for 12 min. Solvent A was composed of 0.1% formic acid (Honeywell) in LC–MS hypergrade water (Supelco). All solvents were sonicated for 10 min before use. The total LC method runtime was 125 min.

Eluting peptides were ionized using nano‐electrospray ionization in positive mode with a capillary voltage of 3.15 kV into a Synapt G2‐Si qTOF mass spectrometer (Waters Corporation) with a cone voltage of 40 V. Trap energy was set at 4 V and transfer energy at 2 V. Transfer collision ramping for high energy MS/MS was from 16 to 50 V before entering the TOF chamber for acquisition between 50 and 2000 Th in positive resolution mode with ion mobility separation (HDMSe) activated across 200 bins, and the wave velocity for the IMS chamber manually set to 1000 m/s. Human Glu‐Fibrinopeptide B was used as the LockMass using a dedicated TaperTip capillary set to 2.30 kV, acquired every 45 s for 5 averaged scans.

Peak picking was done automatically with a maximum allowed charge of +6 using the threshold cutoffs generated using the Uniprot reviewed *Homo sapiens* proteome (acquired May 2025) using Progenesis QI for Proteomics (v.4.0.x). Runs were automatically aligned against a combined pool of samples and normalization was performed using standard settings considering all identified peptide ions. Proteins were identified using the same Uniprot proteome (Figure S1A), allowing 1 missed tryptic cleavage, a maximum protein mass of 600 kDa, fixed carbamidomethylation of cysteines, variable methionine oxidation, a maximum peptide tolerance of 20 ppm, maximum fragment tolerance of 10 ppm, and an FDR cutoff of 4%. Ion matching requirements were at least 1 fragment per peptide, 3 fragments per protein, and 1 peptide per protein.

### 2.4 Data Analysis

Proteins obtained by UPLC–MS/MS with less than 30% of observations were removed. All analyses were performed blinded. Linear models were fitted for each protein, including the covariables of age and gender, in *limma* (RRID:SCR_010943) package in R Studio. The empirical Bayes method was applied to moderate the standard errors and improve statistical power across proteins. All *p*‐values obtained were adjusted for multiple testing using the Benjamini–Hochberg false discovery rate (FDR) correction. Proteins with adjusted *p*‐values (reported directly as *p*‐value) < 0.05 were considered statistically significant and applied to over‐representation analysis.

Both volcano plot and principal component analysis (PCA) were produced with all proteins. Tridimensional PCA was built with three principal components (PC). Furthermore, scores of each PC were obtained for every individual and compared between groups [17]. Those with *p* < 0.05 were applied to Over‐representation analysis using Reactome (RRID:SCR_003485) database in Enrichr (RRID:SCR_001575) website (https://maayanlab.cloud/Enrichr/). Significant pathways were then grouped in their respective four super pathways, encompassing well‐established biological systems for additional analyses. Vector PCA and a general heatmap were built for each super pathway. Differentially expressed (DE) proteins were shown in bar graphs sorted by association to the neutropenia condition.

Protein–Protein‐Interaction (PPI) networks were constructed with the *rbioapi* package. Clusters were identified by Louvain and used to construct the network. Furthermore, we identified the proteins with interactions among clusters and inserted them into EnrichR again to identify the major functionalities of clusters, which were then used to manually annotate (Figure S1B).

## 3 Results

### 3.1 Characterization of the Study Population

In total 20 participants were included (10 patients with chemotherapy‐induced neutropenia and 10 HI), matched for age and gender, selected among patients' data and sample availability. Demographic and clinical characteristics are shown in Table 1. Mean neutrophil and platelet count (0.15 × 10^9^/L; SD = 0.18 and 34.6 × 10^9^/L; SD = 17.4, respectively), confirming that a cohort with severe neutropenia was obtained.

**TABLE 1:** Clinical and demographic characteristics of healthy individuals and patients with neutropenia.

Clinical and demographic characteristics	Healthy individuals (*n* = 10)	Neutropenia (*n* = 10)
Sex (male: female)	5:5	5:5
Age (median, IQR)	51 (41–57)	50 (35–63)
Diagnosis, *n* (%)		
Multiple myeloma	—	6 (60)
Acute leukemia	—	2 (20)
Lymphomas	—	2 (20)
Treatment modality, *n* (%)		
High dose myeloablative CTx	—	9 (90)
High dose CTx for HSCT conditioning	—	1 (10)
ECOG performance status (median, IQR)	—	2 (2–3)
Neutrophils × 10^9^/L (mean ± SD)	NA	0.15 ± 0.18
Platelets × 10^9^/L (mean ± SD)	NA	34.6 ± 17.4
Days after CTx (median, IQR)	—	6 (3–7)
Complications during follow‐up		
Febrile neutropenia (yes: no)	—	8:2
Septic shock (yes: no)	—	1:9
30‐day survival (yes: no)	—	9:1

Abbreviations: CTx, chemotherapy; ECOG, Eastern Cooperative Oncology Group or Electrocorticography; HSCT, hematopoietic stem‐cell transfusion; IQR, interquartile range; NA, not available; SD, standard deviation.

### 3.2 Differentially Expressed Proteins

**FIGURE 1:**
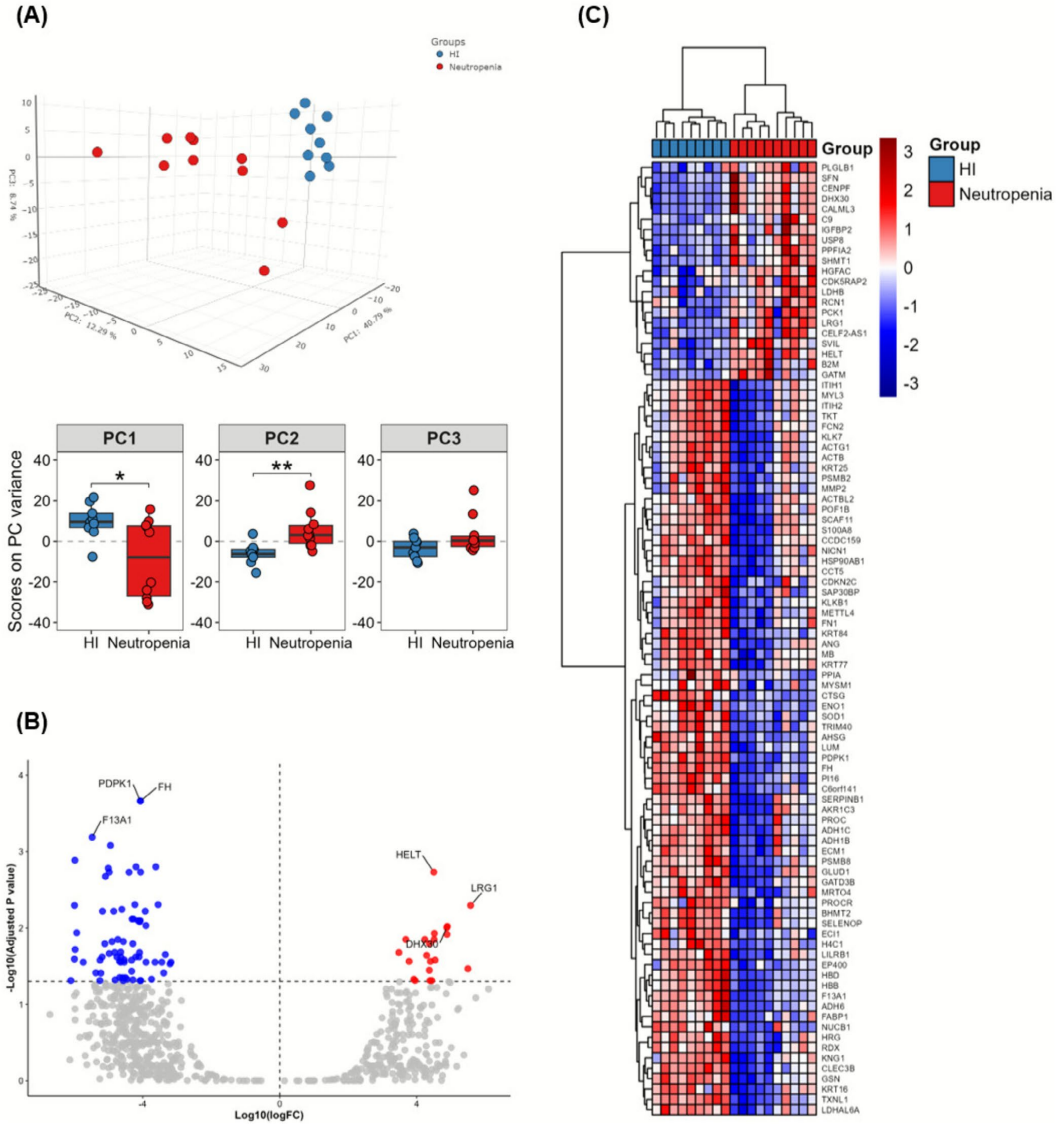
Proteomic signature of severe chemotherapy‐induced neutropenia. (A) Three‐dimensional PCA with all proteins and components that represent around 60% group variance, and Principal Components (PC) signature between both groups. Comparison analysis was made with nonpaired Wilcoxon test. *p < 0.05; **p < 0.01. (B) Volcano plot highlighting differentially expressed proteins. Significance and log_10_ fold change was calculated using a mixed model, corrected by FDR. (C) Heatmap was constructed with 92 differentially expressed proteins found in mixed model.

A total of 762 proteins were identified in all samples. A three‐dimensional PCA was constructed yielding three components that explain little more than 60% of the variation between groups. PC1 explained 40.7% of the total variance in the dataset. When comparing the PC1 scores between groups, the HI group showed significantly higher PC1 scores (*p* < 0.05). Similarly, PC2 (12.29% of variance explained) showed significantly higher scores in the neutropenia group (*p* < 0.01) (Figure 1A).

A total of 92 DE proteins were identified, of which 71 were downregulated and 21 were upregulated in patients with neutropenia (Figure 1B,C). A complete list of down‐ and upregulated pathways is shown in Table S1.

### 3.3 Over‐Representation Analysis of Pathways Based on DE Proteins

**FIGURE 2:**
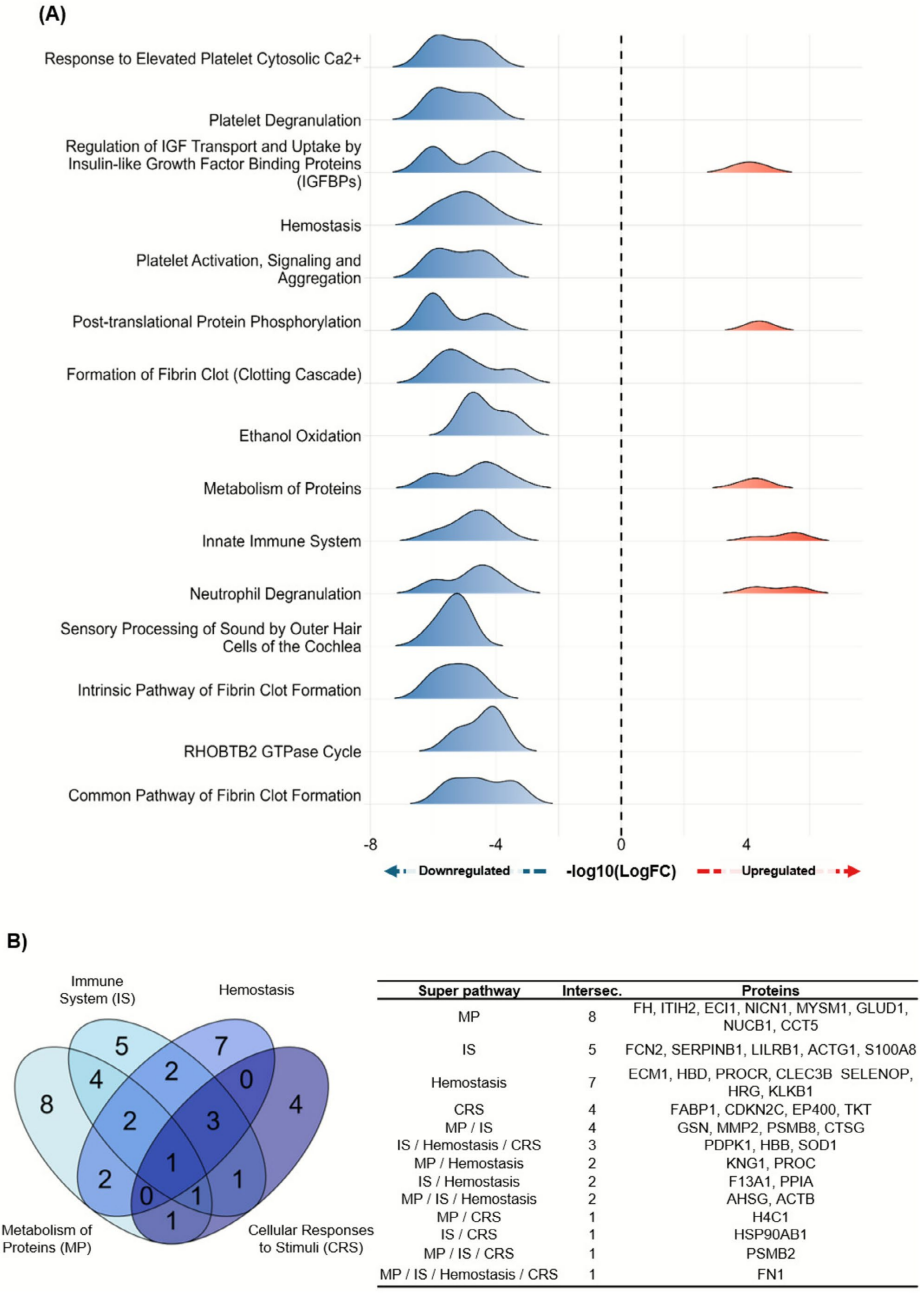
Over‐represented pathways derived from differentially expressed proteins. (A) Pathways identified by in silico functional analysis using all differentially expressed proteins are shown. Down‐ and upregulation in Neutropenia patients are plotted in blue and red respectively, along with logFC changes. (B) Downregulated proteins were inserted in a Venn diagram from the four most significant super pathways found in ORA. On the right, the report for each pathway and intersection is shown as a list. MP had 8 proteins exclusively, while IS had 5, Hemostasis had 7 and CRS had 4. Other proteins were shared between these four pathways. A blue gradient was applied based on super pathways.

All DE proteins were used for over‐representation analysis from which the top 15 most significant pathways were plotted according to their association with the group Neutropenia or HI (Figure 2A). The four most significant “super pathways” which include several related sub pathways were as follows: metabolism of proteins (MP); immune system (IS); hemostasis; and cellular responses to stimuli (CRS). Distribution of DE proteins among these super pathways is shown in a Venn Diagram (Figure 2B). From these four pathways, we focused our discussion on those super pathways with more than 15 modulated (up and/or down) proteins. From this, we found three: (i) related to hemostasis, involving both fibrin formation and platelet activation, which were consistently downregulated in neutropenia; (ii) pathways related to metabolism of proteins; and (iii) immune system presented a more heterogenous expression profile, containing proteins that were both up‐ or downregulated.

#### 3.3.1 Metabolism of Proteins Super Pathway

**FIGURE 3:**
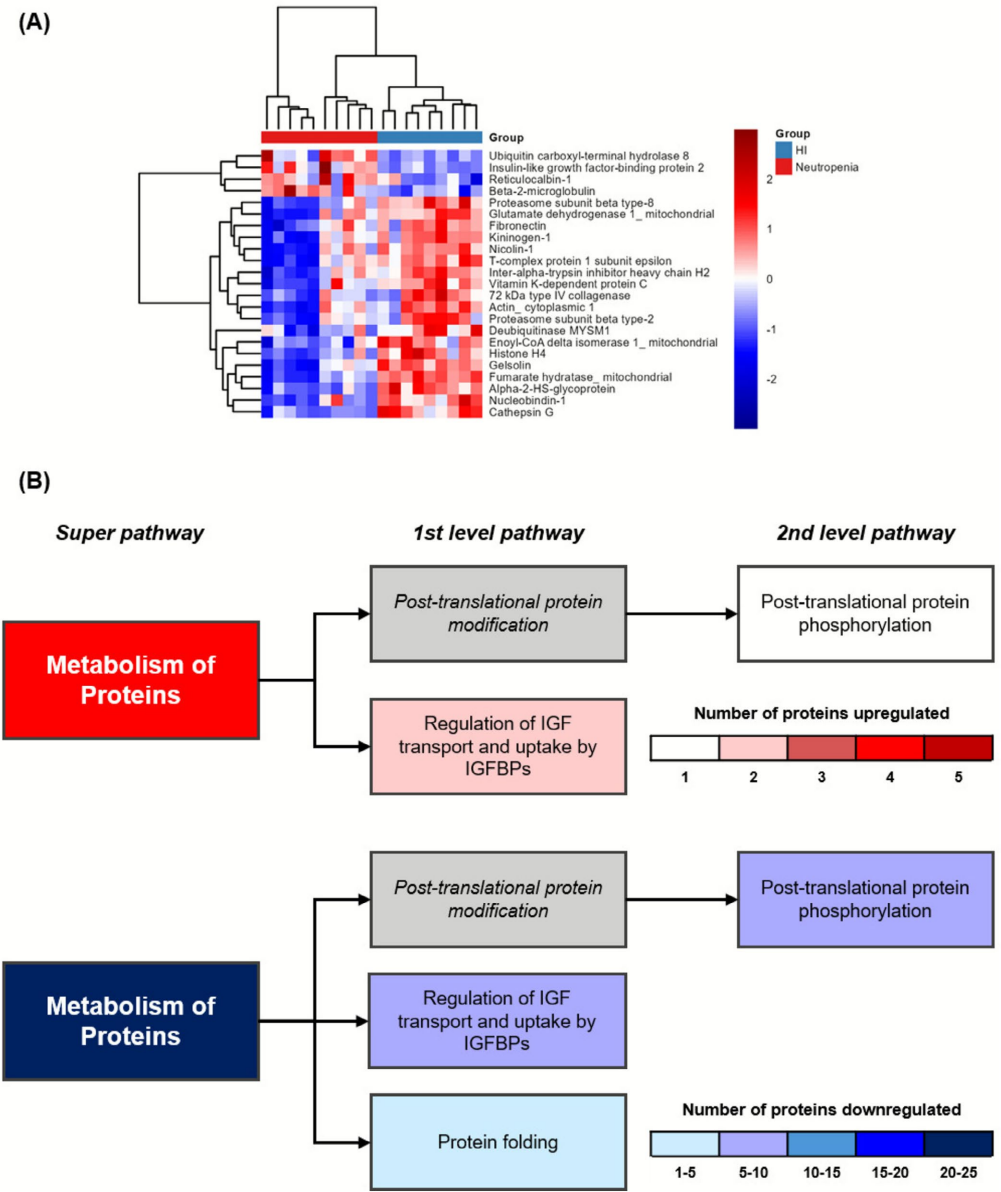
Proteins differentially expressed in the Metabolism of Proteins (MP) super pathway. MP related proteins (*n* = 23) were isolated and analyzed their dynamics for kinetics. (A) Heatmap was constructed with proteins from MP pathway scaled by Z‐score; (B) Schematic overview of pathways and involvement of minor pathways colored by genes involved. Gradient color code was applied to upregulated (red) and downregulated (blue). Pathways in gray were found not significant in EnrichR analysis.

Twenty‐three DE proteins were linked to MP super pathway, of which 4 were upregulated and 19 were downregulated in patients from the Neutropenia group. The four upregulated proteins in patients with neutropenia were Ubiquitin carboxyl‐terminal hydrolase 8 (USP8), insulin‐like growth factor‐binding protein 2 (IGFBP2), reticulocalbin‐1 (RCN1), and Beta‐2‐microglobulin (B2M) (Figure 3A).

We next evaluated which sub‐pathways (1st and 2nd level) were represented based on the signature of DE proteins. Post‐translational protein modification and regulation of IGF transport and uptake by IGFBPs contained both up‐ and downregulated proteins (Figure 3B). In addition, protein folding was also identified as a downregulated pathway.

#### 3.3.2 Immune System Super Pathway

**FIGURE 4:**
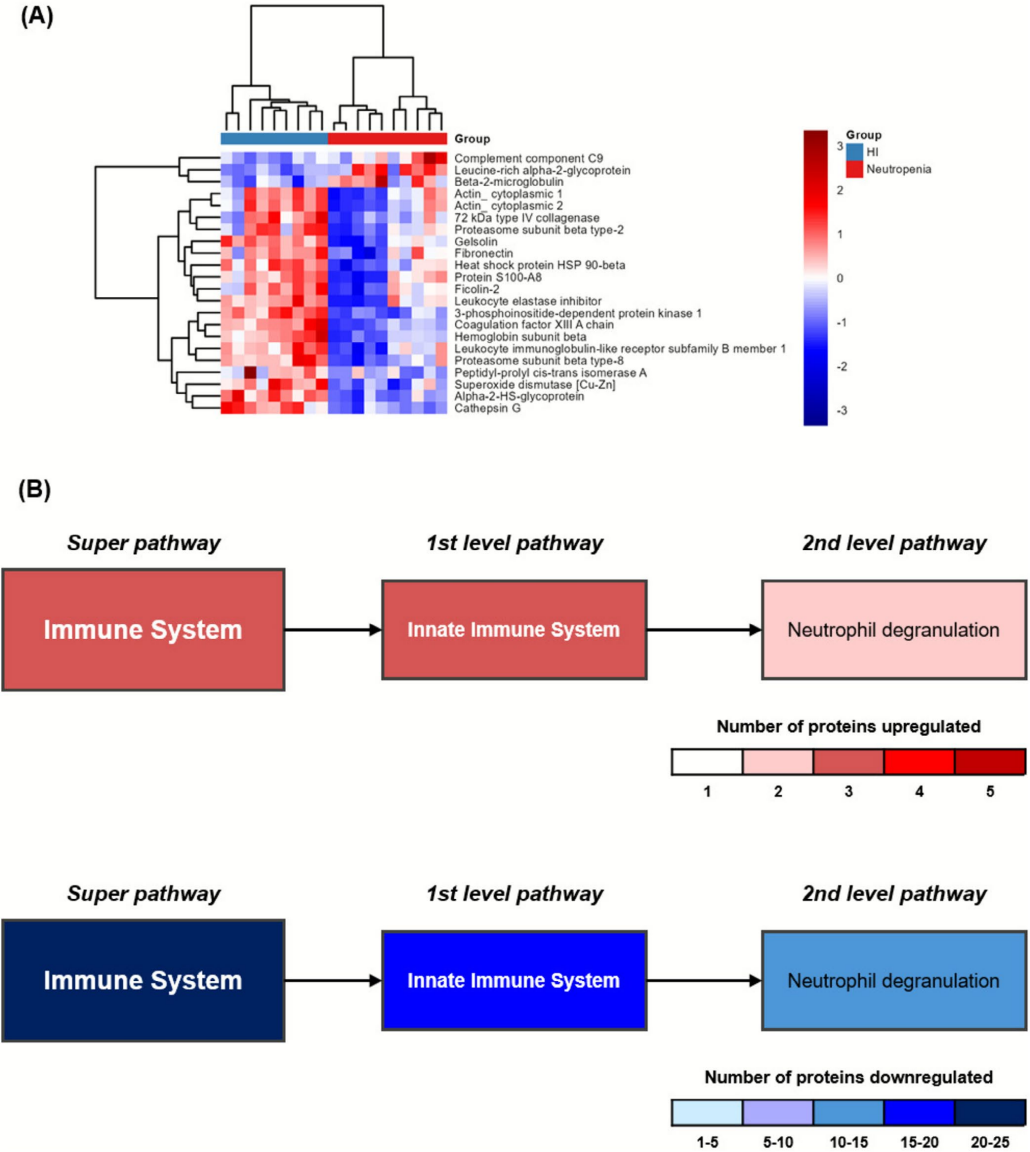
Proteins differentially expressed in Immune System (IS) pathway. IS related proteins (*n* = 22) were isolated and analyzed their dynamics for kinetics. (A) Heatmap was constructed with proteins from IS pathway scaled by Z‐score; (B) Schematic overview of pathways and involvement of minor pathways colored by genes involved. Gradient color code was applied to upregulated (red) and downregulated (blue). Pathways in gray were found not significant in EnrichR analysis.

The immune system pathway was the second bigger pathway, with 22 DE proteins. This pathway was composed of 3 upregulated and 19 downregulated proteins in the Neutropenia group. Complement component C9 (C9), leucine‐rich alpha‐2‐glycoprotein (LRG1), and B2M were found to be upregulated, as shown in Figure 4A. Pathways also highlight the complex impact of chemotherapy‐induced neutropenia on pathways related to both neutrophil function and their capacity to respond to stimuli since proteins linked to neutrophil degranulation were both up‐ or downregulated in these patients (Figure 4B).

#### 3.3.3 Hemostasis Super Pathway

**FIGURE 5:**
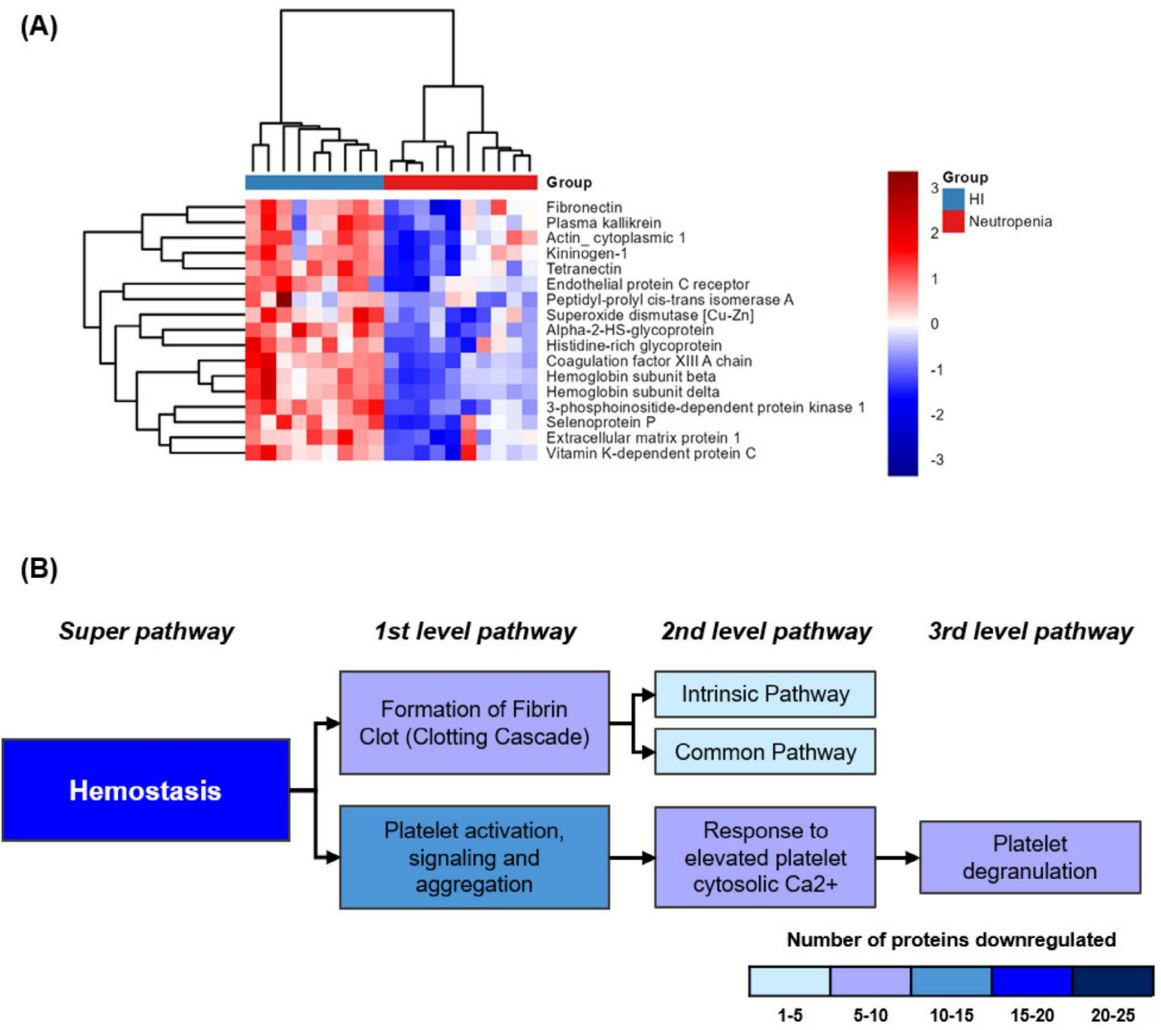
Proteins differentially expressed in Hemostasis pathways. Hemostasis related proteins (*n* = 17) were isolated and analyzed their dynamics for kinetics. (A) Heatmap was constructed with proteins from Hemostasis pathway scaled by *Z*‐score; (B) Schematic overview of pathways and involvement of minor pathways colored by genes involved. There was not seen any protein upregulated in Hemostasis pathway.

In the hemostasis super pathway, all 17 DE proteins were downregulated in the neutropenia group (Figure 5A). Pathway analysis revealed an impact on formation of fibrin clot (clotting cascade), with downregulation of both intrinsic and common pathways. Regarding the pathway of “platelet activation, signaling and aggregation”, the most impacted sub‐pathway was platelet degranulation (Figure 5B).

#### 3.3.4 Cellular Responses to Stimuli (CRS) Super Pathway

In the CRS super pathway, all 11 DE proteins found were also downregulated in the neutropenia group (Figure S2A). Pathway analysis revealed an impact only in the sub pathway of Cellular Response to Chemical Stress (Figure S2B).

### 3.4 Differential Expression of Proteins by Super Pathway

The magnitude of modulation within each super pathway was inferred by kinetics analysis. In the MP super pathway, Fibronectin (FN1) was the top downregulated protein (logFC = −1 259 291; *p* = 0.048), whereas USP8 was the top upregulated (logFC = 27 332; *p* = 0.048), followed by RCN1 (logFC = 25 150; *p* = 0.027), B2M (logFC = 19 488; *p* = 0.022), and IGFB2 (logFC = 5992; *p* = 0.027) (Figure S3A).

Similar to the MP super pathway, FN1 presented the highest downregulation in IS, hemostasis and CRS super pathways. In the IS super pathway LRG1 presented the highest increase (logFC = 374 520; *p* = 0.005), followed by C9 (logFC = 311 844; *p* = 0.003) and B2M (Figure S3B). No upregulated proteins were identified in the Hemostasis or CRS super pathways (Figure S3C,D).

### 3.5 Protein–Protein Interactions

**FIGURE 6:**
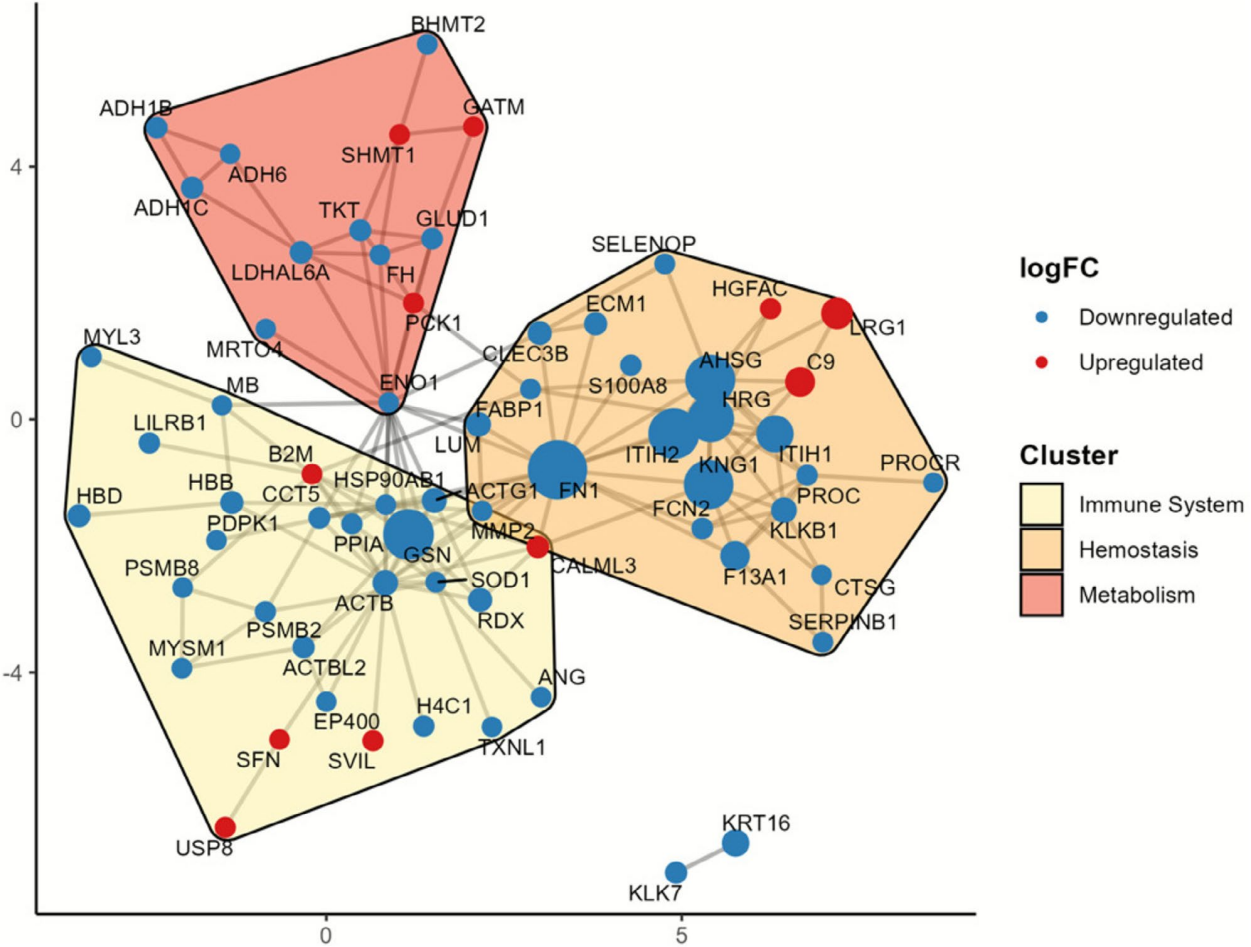
Protein–Protein Interaction (PPI) Network among Neutropenia. DE proteins were applied to PPI Network models and clustered by Louvain. The clusters identified were further inserted in Enrichr and the functionality of the major super pathway annotated manually.

Proteins interactions were plotted in a PPI Network and characterized based on two different patterns: (i) they were clustered by the Louvain Methods, and the most significant super pathway from each cluster was identified and used for manual annotation; (ii) each protein dynamic in a regression model was found, as upregulated or downregulated. Our findings demonstrated that most clusters were related to the immune system, hemostatic or metabolism, as shown in Figure 6. We also observed that proteins with a higher number of interactions were mostly downregulated, although one cannot exclude the occurrence of compensatory pathways.

Despite some upregulated proteins also being shown to mediate functions inside the clusters found, this demonstrates the interactions on proteins and a biological profile connected to functions mostly related to the impact in these three pathways. Although most were downregulated, the dynamics on functionality can propose compensatory models from the biology systems described here.

## 4 Discussion

Transient episodes of severe neutropenia and thrombocytopenia are a frequent complication of the therapy of hematological malignancies and HSCT, rendering patients more prone to sepsis and bleeding. The main contribution of our pilot study was to generate novel data on how myelotoxic chemotherapy affects the plasma proteome that can pave the way for studies that identify the biological mechanisms and/or biomarkers associated with complications of this clinically relevant condition such as febrile neutropenia and sepsis.

Our study focused on patients with severe neutropenia and thrombocytopenia, as evidenced by the very low neutrophil and platelet counts prior to the development of febrile neutropenia or sepsis. The consistent observation of severe neutropenia and thrombocytopenia is a hallmark of the curative treatment of hematological malignancies, which frequently involves a period of chemotherapy‐induced myeloablation. By focusing our analysis in this period, we aimed to access a unique window to observe how the immune and repair systems react to cope with pathogens and tissue damage when these major cellular players are markedly decreased.

A total of 92 DE proteins were identified, with a predominance of downregulated proteins, a trend that is likely associated with the dramatic impact of high‐dose chemotherapy regimens on hematopoiesis and lymphoid development, and that is consistent with a study that explored children with febrile neutropenia using transcriptomics [18]. Notably, we also identified 21 upregulated proteins, which demonstrate that the modulation caused by the cytopenia also involves the upregulation of compensatory pathways. Accordingly, the principal component analysis illustrates the presence of distinct proteomic signatures between our patient population and healthy individuals.

From a more general perspective using a systems biology lens, these 92 DE proteins represented three pathways: immune system (IS), metabolism of proteins (MP), and hemostasis. The first two included mostly downregulated proteins, which we hypothesize to be linked to the anti‐proliferative effect of chemotherapy, which directly affects the proliferation and viability of immune cells. In contrast, the consistent downregulation of hemostasis proteins, involving not only platelet function, but also coagulation processes such as fibrin formation and intrinsic pathway, represent an interesting and novel finding. In recent years, it has become clear that hemostasis and the innate immune system act in concert when coping with pathogens and tissue damage in a process referred to as immunothrombosis [4]. Neutrophils contribute to localized activation of hemostasis by releasing their nuclear content [11], and the contained formation of fibrin network contributes to pathogen and tissue damage compartmentalization [19, 20]. Similarly, platelets contribute to coagulation activation by several mechanisms including the release of procoagulant mediators and the exposure of sites for tissue factor‐mediated coagulation initiation [21, 22], which can potentially explain our observation of a broader downregulation of proteins related to hemostasis, beyond those associated with platelet function. Together, our results highlight a more comprehensive effect of severe neutropenia and thrombocytopenia on immunothrombosis [4, 23]. Since immunothrombosis is a critical process in the host response to pathogens and tissue damage, our data allows us to refine our understanding about the mechanisms of the increased susceptibility of these patients to sepsis and other infectious complications, including the downregulation of hemostasis/immunothrombosis as a contributing factor.

It must be mentioned that the analysis of DE proteins also points to the potential involvement of other pathways, albeit with lower statistical significance, which can be explored by readers using our DE proteins list.

Another perspective to analyze our results is to focus on the individual upregulated proteins, which could be associated with both compensatory pathways triggered by the cytopenias and/or as a response to chemotherapy‐induced damage to tissues and barriers. This individualized approach can also avoid the dependence of enrichment analyses on pre‐curated lists which do not consider novel or more subtle associations between proteins and biological processes. Moreover, considering the relatively low number of individuals from this pilot study, coupled with the baseline inherent heterogeneity of the target population (e.g., baseline diagnosis and chemotherapy regimens), it is fair to assume that upregulated proteins that reached statistical significance in this context likely represent a discrete part of the stereotyped signature that our study aimed to identify, and hence, interesting candidates for future validation studies. Among proteins identified as part of metabolic pathways, the four upregulated proteins were USP8, IGFBP2, RCN1, and B2M, the latter also identified as an immune system‐related protein.

USP8 is a deubiquitination enzyme capable of removing ubiquitin from different substrates, protecting them from degradation, acting in a diverse range of biological processes such as cell proliferation/cell cycle, cancer progression and survival, cell response to stimuli and regulation of protein catabolism/removal of misfolded proteins [24]. It has also been shown to activate the cellular protective NRF2 pathway [25], and to mediate the interferon response in viral infections [26]. Several of these mechanisms are potentially relevant in our patients, allowing us to hypothesize that the upregulation of USP8 is more likely associated with the intense catabolic activity observed after high dose chemotherapy‐associated cell death and tissue barriers disruption. RCN1 is a calcium‐binding protein located in the endoplasmic reticulum lumen [27]. It has also been identified in a variety of tumor cell types including acute leukemias, and in bone marrow endothelial cells [28, 29, 30]. Due to the essential and comprehensive role of calcium in cells as a secondary messenger molecule, RCN1 could be potentially involved in many cell processes such as signal transduction, apoptosis, exocytosis, cytoskeletal dynamics, and cell proliferation, among others. While it is tempting to highlight its expression in bone marrow endothelial cells as a clue to its upregulation in our patients, additional studies are necessary to explore the upregulation of RCN1 in this context. B2M is a component of the class I major histocompatibility complex involved in antigen presentation to the immune system [31]. It is involved in the cytotoxic T‐lymphocyte immune response, but also contributes to the innate immune response by generating antimicrobial peptides [32, 33]. B2M is also involved in amyloid fibril deposition [34]. Clinically, it is used as a biomarker of tumor burden in multiple myeloma [35] and has been shown to associate with worse prognosis in sepsis [36] and during HSCT [37]. We hypothesize that the upregulation of B2M after chemotherapy‐induced neutropenia could represent a compensatory mechanism, and/or a consequence of the intense load of antigens presented to the immune system due to chemotherapy‐mediated tissue damage and intestinal barrier breakdown.

Among upregulated proteins, IGFBP2, LRG1, and C9 present the most intriguing associations with our scientific questions. C9 is a pore‐forming component of the membrane attack complex of the complement system, directly involved in bacterial killing [38, 39]. Several lines of evidence link complement activation to immunothrombosis, such as the demonstration of C9 in platelet‐leukocyte aggregates and on extracellular vesicles derived from these cells [40]. In addition, C5 and C9 have recently been shown to associate with thrombin generation [41]. Finally, an intriguing association has been demonstrated of downregulation of the expression of complement component genes, including C9 and higher neutrophil counts [42] in patients with cardiovascular disease. Together, these data allow one to hypothesize that complement activation may represent a compensatory host response less dependent on the presence of neutrophils and platelets.

Another interesting finding of our study was the upregulation of LRG1. LRG1 is a protein that has been shown to be involved in a variety of biological processes such as angiogenesis, wound healing, immune response, and myeloid cell development [43, 44]. It is constitutively produced and secreted by hepatocytes but has been shown to behave as a biomarker of the activity of acute and chronic inflammatory conditions including sepsis [45], cardiovascular diseases [46], among others. LRG1 is expressed during granulocyte differentiation and is involved in myeloid maturation [47]. In recent years, evidence emerged linking LRG1 to processes such as modulation of the M2 macrophage phenotype [48], myocardial protection after acute myocardial infarction [49] and regulation of myelopoiesis. Interestingly, the latter was mediated by antagonization of the inhibitory effect of TGFb1 on CD34+ and myeloid progenitors [50]. The same group demonstrated that LRG1 is packaged into the granule compartment of human neutrophils and secreted upon neutrophil activation [50]. Notably, upregulation of LRG1 expression was also identified in a cohort of acute myeloid leukemia patients (AML) experiencing neutropenia [51]. We believe that this evidence allows us to hypothesize that LRG1 might be an important element of host response to chemotherapy‐induced neutropenia, whose biological and potential therapeutic implications warrant additional studies.

The last upregulated protein that we highlight is IGFBP2, a secreted protein that regulates the availability of insulin growth factors IGF1 and IGF2, thus modulating several processes mediated in this important endocrine function such as signaling, cell proliferation, and survival in various cell‐specific contexts. While not frequently mutated in cancers, levels of IGFBP2 and other IGFBP proteins have been associated with the prognosis of several cancers, including acute myeloid leukemia, possibly because they mediate processes that are co‐opted by cancer cells [52, 53]. Most interestingly for our context, it has recently been shown that IGFBP2 is capable of mediating the production of hematopoietic progenitor cells in vitro, possibly by remodeling metabolic activity during in vitro endothelial to hematopoietic transition [54], a finding that is in line with a prior observation that IGFBP2 was capable of enhancing ex vivo platelet production [55]. Interestingly, upregulation of the IGF/IGFBP pathway has also been demonstrated after myelotoxic insults such as chemotherapy for childhood leukemia [56] and experimental total body irradiation in animal models [57]. Accordingly, the upregulation of IGFBP2 in our study could illustrate one of the most early detectable elements of the host response to myelotoxicity.

Finally, we also performed a cluster analysis based on protein–protein interactions for both down‐ and upregulated proteins. Results from these analyses were largely in line with results from pathway enrichment analysis. Downregulated proteins were clustered in protein networks involved in fibrin formation/clot cascade, platelet function and proliferation, as well as in more broader processes such as cytoskeletal dynamics, which are involved in both platelet function and hematopoietic cell differentiation/proliferation. Two different processes were identified, namely degradation of extracellular matrix and oxidation/metabolism, which are ubiquitous biological processes that, in the context of our study, could be related to tissue damage, disruption of tissue barriers, and cell death. While the number of clusters derived from upregulated proteins was much lower, they provide additional support to the concept that complement activation is present in these patients. We acknowledge that other pathways are also present within the biological background of Cx‐induced neutropenia, which must be explored deeply on how they impact system biology.

Our study has limitations that need to be acknowledged. First, this study was designed as a discovery proteomics analysis and did not include experimental validation of the enriched pathways. Future studies are warranted to confirm or refute these associations, as well as to explore their biological relevance: Second, it was based on a relatively low number of individuals so that (i) more subtle differences in the proteomic signature might have been missed, and (ii) patient heterogeneity related to baseline diagnosis and chemotherapy regimen might have influenced our results. However, it should be noted that the study was designed as a pilot, exploratory and hypothesis‐generating step aimed to generate an open database of DE proteins that can pave the way for more detailed studies designed to confirm the biological relevance of individual findings by mechanistic functional studies and/or in clinical studies with larger sample size. On the other hand, we believe that given the relatively low “*n*” and baseline heterogeneity, it is fair to consider DE proteins that stood out in this stringent experimental context, likely representing true and relevant changes of the human proteome caused by this specific and clinically relevant insult and, hence, attractive topics for these necessary confirmatory studies.

In conclusion, chemotherapy‐induced severe neutropenia is associated with a broad set of alterations in the human proteome, involving mostly the downregulation of proteins and pathways involved in hemostasis, immune system, and protein metabolism, pointing to a compromise of the individual's host response to pathogens and tissue damage. In addition, by exploring the few upregulated pathways, we were able to identify candidate proteins that could represent early compensatory mechanisms by which the host responds to bone marrow and systemic toxicity induced by intensive chemotherapy regimens. Future studies are warranted to explore the biological and clinical relevance of these candidate proteins and pathways in this population.

## Author Contributions

L.Q.S., A.L.S.‐J., L.C.S.‐C., and B.J.S. conceived, designed and analyzed the data; L.Q.S. and I.T.B.‐J. recruited the participants; L.Q.S., L.C.S.‐C., and B.J.S. acquired and processed the preliminary data; E.T.A., B.K.L.D., M.P.C., T.M.S., D.M.‐S. and E.V.D.P. interpreted the data; A.L.S.‐J., and E.V.D.P. drafted the manuscript. All authors revised the final draft.

## Funding

This work was supported by Fundação de Amparo à Pesquisa do Estado de São Paulo (FAPESP), #2021/12945‐6, #2025/04429‐9, #2023/08178‐5; Coordenação de Aperfeiçoamento de Pessoal de Nível Superior (CAPES); Conselho Nacional de Desenvolvimento Científico e Tecnológico (CNPq); Fundação de Amparo à Pesquisa do Estado de São Paulo (FAPESP), #2022/13216‐0.

## Conflicts of Interest

The authors declare no conflicts of interest.

## Supporting information


**Figure S1:** fsb271517‐sup‐0001‐FigureS1.png.

**Figure S2:** fsb271517‐sup‐0002‐FigureS2.png.

**Figure S3:** fsb271517‐sup‐0003‐FigureS3.png.

**Table S1:** fsb271517‐sup‐0004‐TableS1.docx.

## Data Availability

The data that support the findings of this study are available on request from the corresponding author. The data are not publicly available due to privacy or ethical restrictions.
