# Supplementary figures and images for: Plasma Proteomic Profile of Chemotherapy‐Induced Severe Neutropenia: A Pilot Discovery Phase Study
Source: FASEB J. 2026 Feb 1;40(3):e71517. doi: 10.1096/fj.202503947RR (PMC12862286; doi:10.1096/fj.202503947RR)

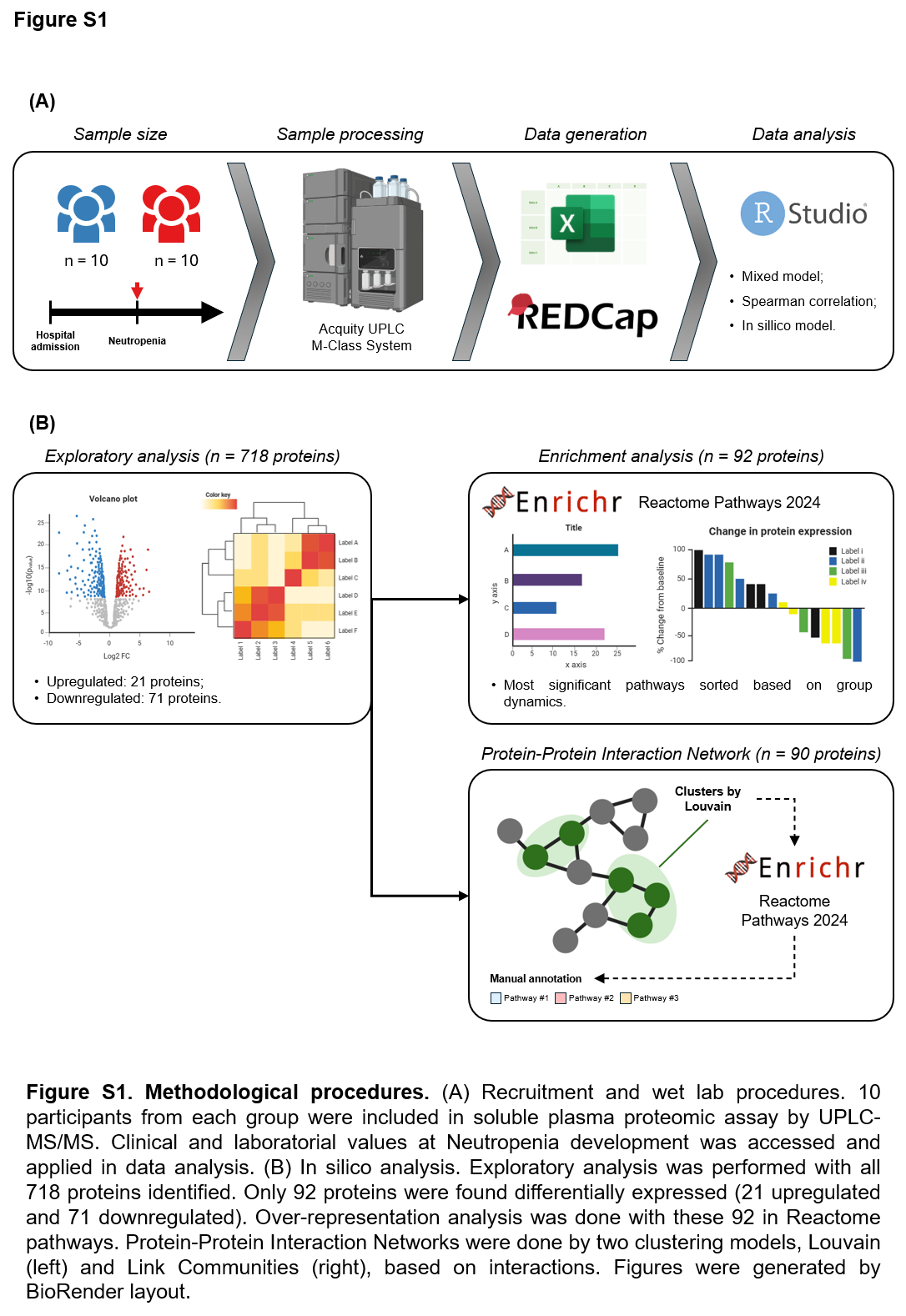

Supplement: Supplementary file 1 — Figure S1: fsb271517‐sup‐0001‐FigureS1.png. [file FSB2-40-e71517-s001.png]

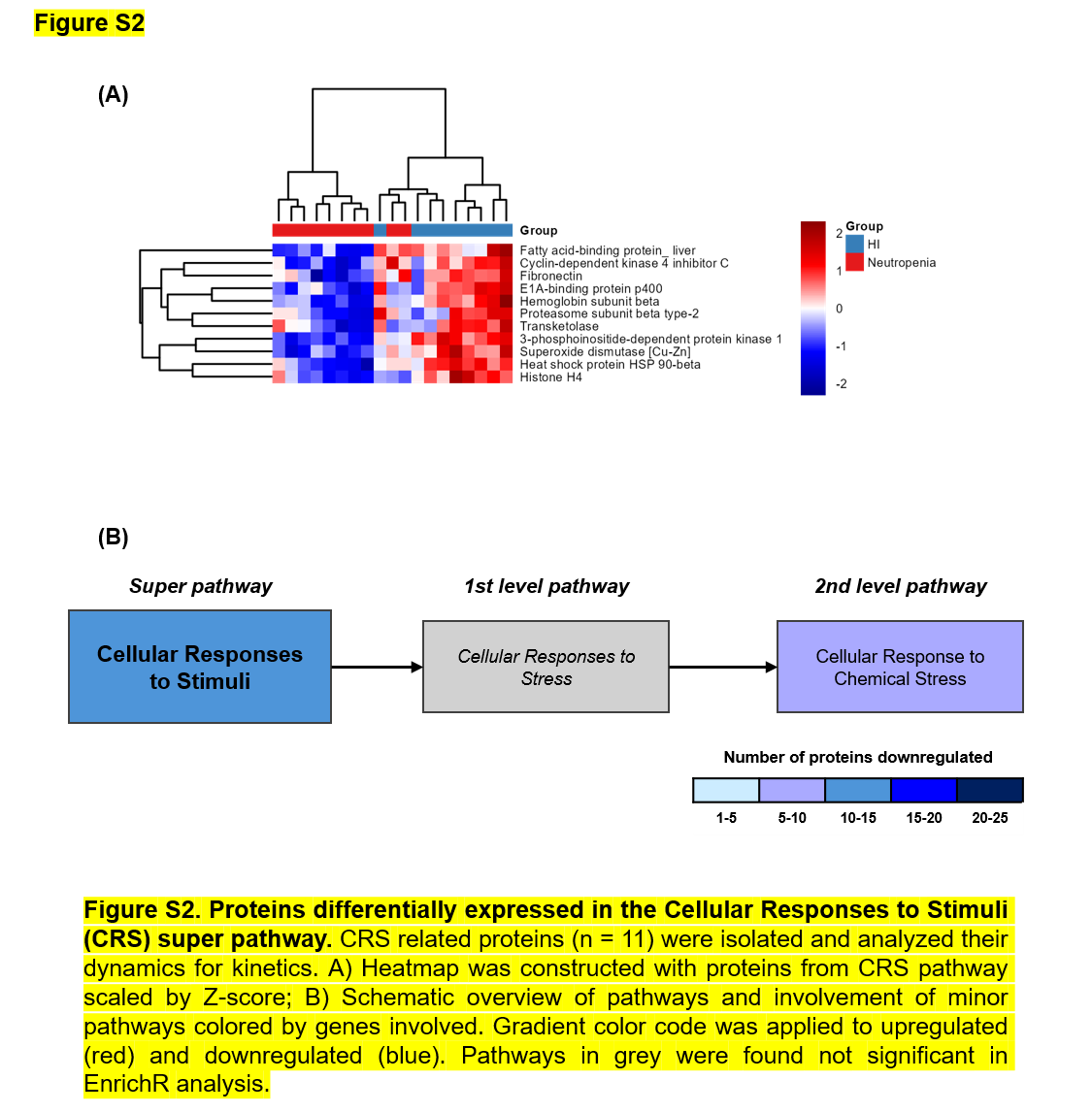

Supplement: Supplementary file 2 — Figure S2: fsb271517‐sup‐0002‐FigureS2.png. [file FSB2-40-e71517-s002.png]

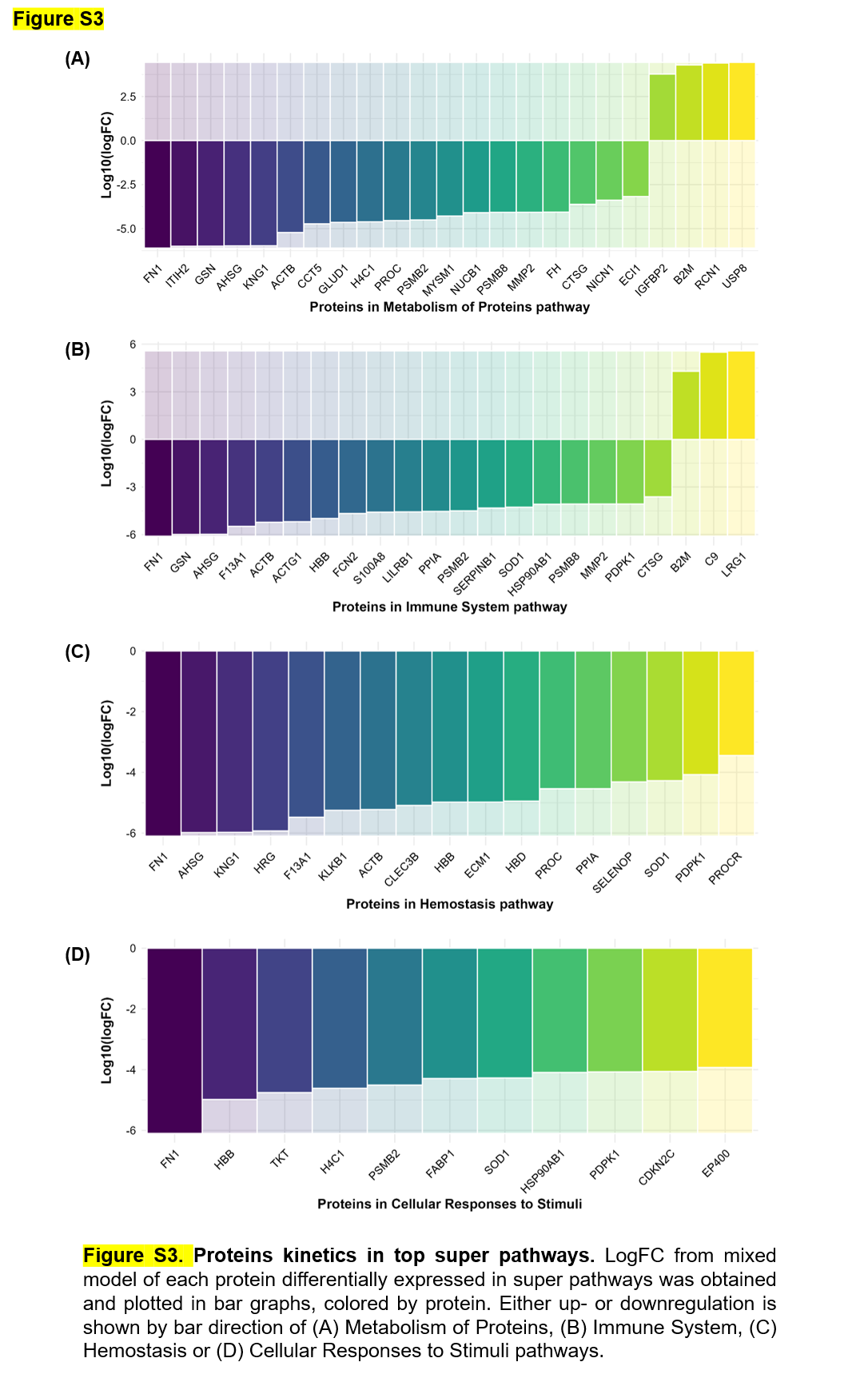

Supplement: Supplementary file 3 — Figure S3: fsb271517‐sup‐0003‐FigureS3.png. [file FSB2-40-e71517-s004.png]
