# Supplementary material for: Plasma Proteomic Profile of Chemotherapy‐Induced Severe Neutropenia: A Pilot Discovery Phase Study
Source: FASEB J. 2026 Feb 1;40(3):e71517. doi: 10.1096/fj.202503947RR (PMC12862286; doi:10.1096/fj.202503947RR)
Supplement: Supplementary file 4 — Table S1: fsb271517‐sup‐0004‐TableS1.docx. [file FSB2-40-e71517-s003.docx]

**Table S1:** Analysis of differentially expressed proteins in Neutropenia group, with respective logFC and p value.

| **Gene** | **Protein name** | **logFC** | **p value** |
| --- | --- | --- | --- |
| LRG1 | Leucine-rich alpha-2-glycoprotein | 374520.29 | 0.0051 |
| C9 | Complement component C9 | 311844.39 | 0.0342 |
| DHX30 | ATP-dependent RNA helicase DHX30 | 78209.14 | 0.0095 |
| CENPF | Centromere protein F | 76623 | 0.0121 |
| CALML3 | Calmodulin-like protein 3 | 75677.58 | 0.0099 |
| LDHB | L-lactate dehydrogenase B chain | 34343.73 | 0.0263 |
| HGFAC | Hepatocyte growth factor activator | 32801.36 | 0.0118 |
| SVIL | Supervillin | 32221.81 | 0.0142 |
| HELT | Hairy and enhancer of split-related protein HELT | 31555.73 | 0.0019 |
| USP8 | Ubiquitin carboxyl-terminal hydrolase 8 | 27332.84 | 0.0488 |
| RCN1 | Reticulocalbin-1 | 25150.8 | 0.0274 |
| PLGLB1 | Plasminogen-like protein B | 24530.36 | 0.0488 |
| PCK1 | Phosphoenolpyruvate carboxykinase cytosolic [GTP] | 23613.37 | 0.0161 |
| CDK5RAP2 | CDK5 regulatory subunit-associated protein 2 | 23382.34 | 0.0358 |
| B2M | Beta-2-microglobulin | 19488.04 | 0.0228 |
| SHMT1 | Serine hydroxymethyltransferase cytosolic | 17227.43 | 0.0142 |
| GATM | Glycine amidinotransferase mitochondrial | 8908.7 | 0.0487 |
| SFN | 14-3-3 protein sigma | 8270.35 | 0.0469 |
| IGFBP2 | Insulin-like growth factor-binding protein 2 | 5992.57 | 0.0274 |
| PPFIA2 | Liprin-alpha-2 | 4799.62 | 0.0141 |
| CELF2-AS1 | Putative uncharacterized protein CELF2-AS1 | 3009.67 | 0.021 |
| ECI1 | Enoyl-CoA delta isomerase 1 mitochondrial | -1499.17 | 0.0281 |
| MRTO4 | mRNA turnover protein 4 homolog | -1593.37 | 0.0295 |
| GATD3B | Putative glutamine amidotransferase-like class 1 domain-containing protein 3B mitochondrial | -2191.17 | 0.0223 |
| NICN1 | Nicolin-1 | -2439.3 | 0.0387 |
| PROCR | Endothelial protein C receptor | -2773.49 | 0.0281 |
| ADH6 | Alcohol dehydrogenase 6 | -3552.24 | 0.0049 |
| CTSG | Cathepsin G | -4164.8 | 0.0016 |
| SAP30BP | SAP30-binding protein | -4383.85 | 0.0281 |
| MB | Myoglobin | -5531.13 | 0.0469 |
| CCDC159 | Coiled-coil domain-containing protein 159 | -8027.5 | 0.0281 |
| ENO1 | Alpha-enolase | -8084.09 | 0.0093 |
| EP400 | E1A-binding protein p400 | -8466.59 | 0.006 |
| CDKN2C | Cyclin-dependent kinase 4 inhibitor C | -11492.37 | 0.0487 |
| TXNL1 | Thioredoxin-like protein 1 | -11557.47 | 0.0019 |
| FH | Fumarate hydratase mitochondrial | -11574.11 | 0.0002 |
| PDPK1 | 3-phosphoinositide-dependent protein kinase 1 | -11834.57 | 0.0002 |
| MMP2 | 72 kDa type IV collagenase | -11842.88 | 0.0488 |
| PSMB8 | Proteasome subunit beta type-8 | -11885.04 | 0.008 |
| HSP90AB1 | Heat shock protein HSP 90-beta | -12318.67 | 0.0083 |
| NUCB1 | Nucleobindin-1 | -12419.16 | 0.021 |
| MYL3 | Myosin light chain 3 | -12785.86 | 0.0239 |
| TRIM40 | E3 ubiquitin ligase TRIM40 | -13688.92 | 0.008 |
| PI16 | Peptidase inhibitor 16 | -15728.48 | 0.0016 |
| ANG | Angiogenin | -15955.61 | 0.0239 |
| BHMT2 | S-methylmethionine--homocysteine S-methyltransferase BHMT2 | -16981.62 | 0.0049 |
| SOD1 | Superoxide dismutase [Cu-Zn] | -18774.31 | 0.0076 |
| MYSM1 | Deubiquitinase MYSM1 | -19387.79 | 0.0476 |
| FABP1 | Fatty acid-binding protein liver | -19538.8 | 0.0161 |
| SELENOP | Selenoprotein P | -20513.45 | 0.0076 |
| POF1B | Protein POF1B | -21107.19 | 0.0368 |
| SERPINB1 | Leukocyte elastase inhibitor | -21235.09 | 0.0264 |
| C6orf141 | Uncharacterized protein C6orf141 | -25080.64 | 0.0019 |
| KRT25 | Keratin type I cytoskeletal 25 | -28918.35 | 0.0469 |
| PSMB2 | Proteasome subunit beta type-2 | -32045.24 | 0.0447 |
| PPIA | Peptidyl-prolyl cis-trans isomerase A | -34679.66 | 0.0263 |
| PROC | Vitamin K-dependent protein C | -34699.9 | 0.0281 |
| LILRB1 | Leukocyte immunoglobulin-like receptor subfamily B member 1 | -36840.88 | 0.0151 |
| AKR1C3 | Aldo-keto reductase family 1 member C3 | -37114.33 | 0.0488 |
| S100A8 | Protein S100-A8 | -38766.07 | 0.0447 |
| H4C1 | Histone H4 | -40780.16 | 0.0057 |
| METTL4 | N(6)-adenine-specific methyltransferase METTL4 | -43655.94 | 0.0239 |
| GLUD1 | Glutamate dehydrogenase 1 mitochondrial | -43937.26 | 0.0281 |
| ADH1B | All-trans-retinol dehydrogenase [NAD(+)] ADH1B | -44041.22 | 0.0207 |
| SCAF11 | Protein SCAF11 | -47083.68 | 0.0445 |
| FCN2 | Ficolin-2 | -47240.54 | 0.0274 |
| CCT5 | T-complex protein 1 subunit epsilon | -53334.27 | 0.0142 |
| TKT | Transketolase | -56963.23 | 0.0239 |
| ACTBL2 | Beta-actin-like protein 2 | -63978.11 | 0.0477 |
| KLK7 | Kallikrein-7 | -65838.6 | 0.0209 |
| ADH1C | Alcohol dehydrogenase 1C | -70309.02 | 0.006 |
| LDHAL6A | L-lactate dehydrogenase A-like 6A | -78881.3 | 0.0151 |
| HBD | Hemoglobin subunit delta | -87866.09 | 0.0008 |
| ECM1 | Extracellular matrix protein 1 | -95529.11 | 0.0217 |
| HBB | Hemoglobin subunit beta | -95598.39 | 0.0019 |
| KRT84 | Keratin type II cuticular Hb4 | -101614.63 | 0.0016 |
| CLEC3B | Tetranectin | -122738.14 | 0.0021 |
| RDX | Radixin | -128136.76 | 0.0161 |
| LUM | Lumican | -149720.34 | 0.006 |
| ACTG1 | Actin cytoplasmic 2 | -153318.1 | 0.0239 |
| ACTB | Actin cytoplasmic 1 | -167287.98 | 0.0389 |
| KLKB1 | Plasma kallikrein | -176371.72 | 0.0264 |
| KRT77 | Keratin type II cytoskeletal 1b | -178708.3 | 0.0488 |
| KRT16 | Keratin type I cytoskeletal 16 | -233884.36 | 0.0386 |
| F13A1 | Coagulation factor XIII A chain | -300523.32 | 0.0006 |
| ITIH1 | Inter-alpha-trypsin inhibitor heavy chain H1 | -545848.62 | 0.0281 |
| HRG | Histidine-rich glycoprotein | -848057.14 | 0.0116 |
| KNG1 | Kininogen-1 | -943934.97 | 0.0191 |
| AHSG | Alpha-2-HS-glycoprotein | -958731.59 | 0.0013 |
| GSN | Gelsolin | -981836.78 | 0.0051 |
| ITIH2 | Inter-alpha-trypsin inhibitor heavy chain H2 | -987795.5 | 0.0256 |
| FN1 | Fibronectin | -1259291.19 | 0.0488 |
